# The Impact of Alexithymia on Treatment Response in Psychiatric Disorders: A Systematic Review

**DOI:** 10.3389/fpsyt.2020.00311

**Published:** 2020-04-16

**Authors:** Federica Pinna, Mirko Manchia, Pasquale Paribello, Bernardo Carpiniello

**Affiliations:** ^1^ Section of Psychiatry, Department of Medical Sciences and Public Health, University of Cagliari, Cagliari, Italy; ^2^ Unit of Clinical Psychiatry, University Hospital of Cagliari, Cagliari, Italy; ^3^ Department of Pharmacology, Dalhousie University, Halifax, NS, Canada

**Keywords:** depression, longitudinal, personality disorders, systematic review, eating disorders

## Abstract

Treatment of psychiatric disorders relies heavily on a trial and error approach, often prolonging the time required to obtain symptomatic improvements. The identification of reliable predictors of treatment response is instrumental to enact an individualized approach. Alexithymia represents a personality trait reflecting an intrinsic difficulty in recognizing the emotional components of subjective experiences. Thus, its modulating role on treatment outcome has gathered substantial attention during the past years. In the present paper, we aimed at exploring the available evidence for Alexithymia role in influencing the treatment outcome on a wide range of psychiatric conditions by means of a systematic review.

**Data Source:**

We performed a systematic review in Medline and Scopus, augmented by tracking the reference list of the pertinent articles.

**Inclusion Criteria:**

To be included in this review, research studies had to assess alexithymia impact on a treatment intervention delivered to manage a primary psychiatric disorder.

**Study Evaluation and Data Synthesis:**

After removing duplicates, titles were screened first, then abstracts, and last full texts were read, eventually leading to the inclusion or exclusion of the papers according to the criteria established before the online search. Then results of the search were downloaded in.xml format and uploaded in Rayyan, a free web software, that helps expedite the initial screening of abstracts and titles using a process of semi-automation while incorporating a high level of usability. After uploading, screening of the literature was performed in blind by two investigators. Disagreement between reviewers was resolved by joint discussion with a third senior investigator. The quality of evidence was assessed using the Newcastle Ottawa Scale. Thereafter, the data considered relevant was extracted and synthetized in this paper.

**Results:**

Our search yielded a total of 30 articles dealing with a wide range of psychiatric conditions and exploring both pharmacological and psychotherapeutic interventions. Several lines of evidence suggest a complex role for alexithymia in influencing the psychiatric treatment outcome, further underscoring the need for additional research in this area to better address the existing knowledge gaps.

## Introduction

Psychiatric disorders exact a large toll on society at a global level (1, 2). The epidemiological figure in 2016 showed that more than one billion people worldwide were affected by mental or addictive disorders making up about 16% of the world’s population (2). This determines the very high levels of disability exemplified by the 162.5 million Disability-Adjusted Life Years lost in 2016 (2). In turn, this substantial burden of illness is responsible for the enormous socio-economic costs associated with psychiatric disorders (3).

In this context, reducing the burden of psychiatric illness is vital. Several approaches, including those at large scale, have shown their effectiveness in decreasing the impact of these severe chronic disorders (4, 5). These include interventions that can be implemented in parenting, at schools, at the workplace, in older age, and that focus, among the others, on the innovative use of technological platforms to enhance access, cut costs, and reduce stigma (5). However, direct interventions on psychopathological symptoms, either pharmacological or non-pharmacological, remain the backbone in the clinical management of psychiatric disorders. For instance, historical trends showed that the advent of antipsychotic treatment improved significantly the clinical outcome of patients affected by schizophrenia (6). Yet, the prevalence of common psychiatric disorders has remained unchanged in the last decades (7), suggesting that, even if psychological and pharmacological treatment are effective, several factors might reduce their capacity of decreasing their burden.

In this context, the search for moderators of treatment response has received much attention in the past years. The identification of these factors (clinical and/or biological) might ideally lead to accurate predictive models of response to treatment. Indeed, machine learning algorithms were able to identify relatively accurate predictive models of resistance to antidepressant treatments relying solely on patient self-reported measures (8). Of interest, some clinical factors, such as low energy or presence of psychosis, were among the contributors to the risk of treatment resistance (8). Acting on factors that might limit the effectiveness of treatments, either with primary or secondary/tertiary preventive strategies, might decrease the burden of these severe chronic disorders.

In this context, alexithymia represents a promising candidate. Since its introduction as a psychological construct (9), research has focused on the delineation of its clinical (phenotypic), neurophysiological, neurobiological, and genetic underpinnings (10, 11). These investigation have helped in delineating the developmental trajectory of alexithymia, with the recognition that there might be an etiological heterogeneity leading to the manifestation of alexithymia (12). Indeed, Messina et al. have postulated that at least three forms, primary, secondary, and organic, might characterize alexithymia (12). The presence of contributing factors to the development of alexithymia acquires importance in light of the possibility of reducing its detrimental impact on the effectiveness of treatments. Indeed, the literature shows that alexithymia is a potent predictor of resistance to treatment, and this effect extends beyond the area of psychiatric disorders as testified by the evidence gathered in somatic disorders such as gastrointestinal (13) and dermatologic (14) conditions, among the others. Furthermore, alexithymia can be a precipitating factor of suicidal behavior in patients affected by psychiatric disorders (15–17).

In this manuscript, we aim to perform a qualitative assessment of the literature on the moderating role of alexithymia on psychological and pharmacological interventions in common psychiatric disorders.

Our group has previously investigated the impact of alexithymia on the treatment of eating disorders (18), highlighting: 1) its significant role in modulating the effectiveness of interventions, and 2) that alexithymia levels remain often elevated even in the presence of symptomatologic relief in other dimensions. Building on this work, we expanded the systematic search updating the results on eating disorders and summarizing findings for each diagnostic category.

### Aim

As summarized above, there is consistent evidence pointing to a role of alexithymia in moderating treatment response. It is conceivable that higher levels of alexithymia before treatment initiation might reduce the response to the intervention. In addition, the impact of alexithymia could be more prominent in specific diagnostic categories (for instance, anxiety or eating disorders, than in others, such as psychosis). In this context, we performed a systematic review of the available literature with the aim of clarifying to what extent alexithymia exerts its moderating role on treatment response.

## Methods

### Systematic Assessment of the Literature

**Figure 1:**
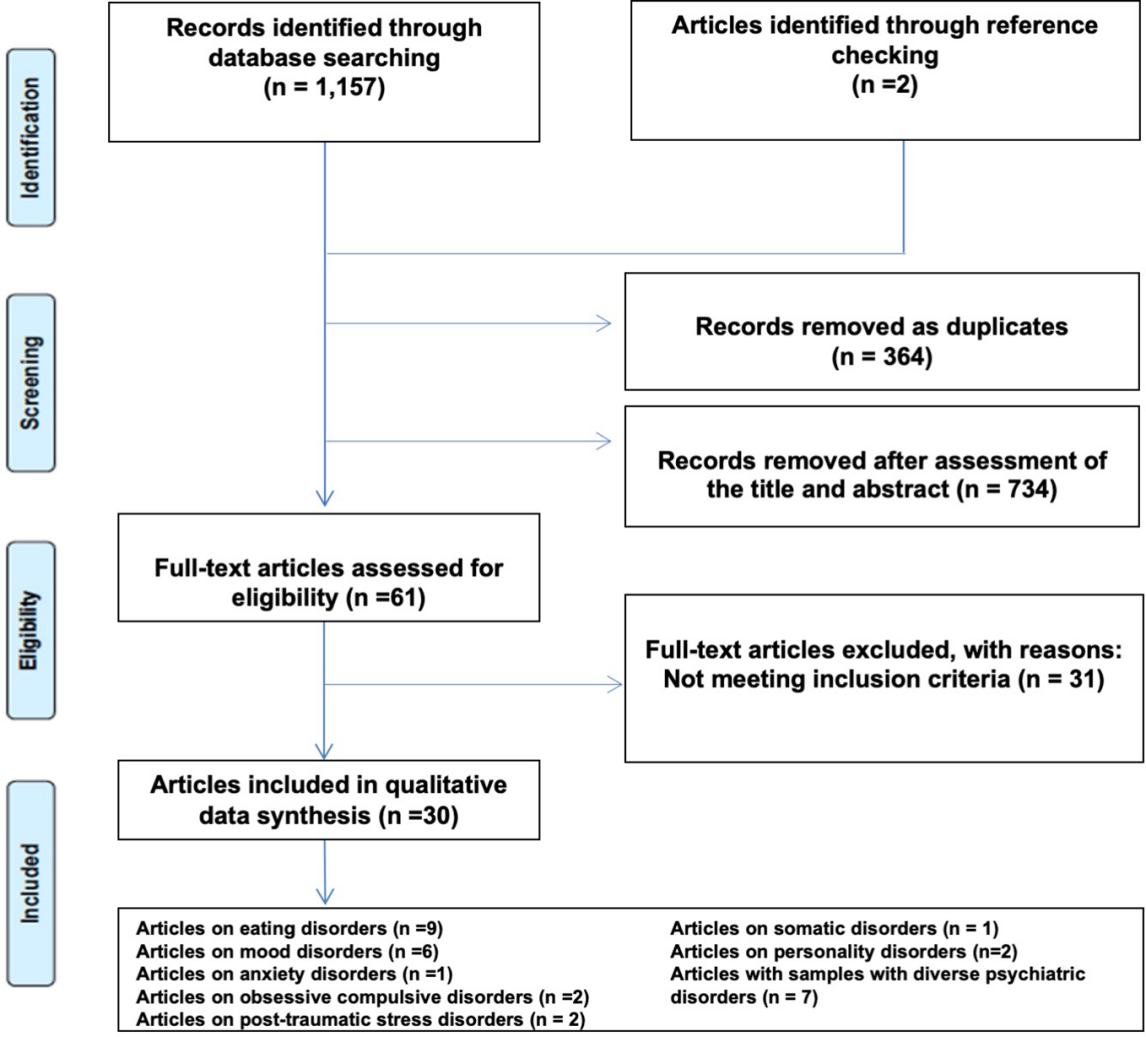
PRISMA flowchart of the study selection.

We performed a systematic review in Medline and Scopus according to Preferred Reporting Items for Systematic Reviews and Meta-Analyses (19) (**Figure 1**) using the following search string: “Alexithymia” AND (“treatment” OR “drug” OR “pharmacotherapy”). The search was performed on October 28, 2019. This search strategy was augmented by identifying additional original reports by tracking citations from reference lists of included articles. After removing duplicates, titles were screened first, and those clearly not in line with the purpose of the review were excluded. Then abstracts were assessed, and last full texts were read, eventually leading to the inclusion or exclusion of the papers, according to the criteria established before the online search. The systematic review of the literature was performed on Medline and Scopus. Then results of the search were downloaded in.xml format and uploaded in Rayyan, a free web software, that helps expedite the initial screening of abstracts and titles using a process of semi-automation while incorporating a high level of usability (20). After uploading, screening of the literature was performed in blind by two investigators (MM and PP). Disagreement between reviewers was resolved by joint discussion with a third senior investigator (FP). The quality of evidence was assessed using the Newcastle Ottawa Scale (NOS) (21).

### Inclusion and Exclusion Criteria

To be included in the review, research studies had to: (a) deal with patients affected by a primary psychiatric disorder (excluding substance use disorders), without limits of age; (b) involve the application of any kind of pharmacological or non-pharmacological treatment; (c) assess alexithymia and the impact of alexithymia on a treatment intervention using a validated standardized assessment tool; (d) be written in English. We excluded reviews and meta-analyses of the literature, case reports, and case series from the systematic appraisal.

### Data Extraction

The following data considered relevant for the systematic search were extracted from each study and tabulated in a data management software: authors, year of publication, study objectives, study design, clinical methodology, assessment tool of alexithymia, type of treatment, diagnostic assessment tools, diagnosis, findings.

## Results

### Literature Search and Selection of Papers

Our systematic search identified 1,157 papers (440 from Medline and 717 from Scopus). 2 papers were added through accurate check of reference lists. After removing 364 duplicates, 795 papers were screened for an initial assessment. We then excluded 734 papers through the assessment of the title and abstract content. The PRISMA selection process is illustrated in **Figure 1**.

### Quality Assessment

The NOS scale average score for the included studies was 3.7 (standard deviation, ± 1.3). The assessment scores are available in the **Supplementary Table**.

### Assessment Tools of Alexithymia

The majority of studies employed the Toronto Alexithymia Scale 20 items (TAS-20) (22, 23) and its older 26-item version (24). One study applied the Alexithymia Provoked Response Questionnaire APRQ (25).

### Included Studies

Thirty studies were included in the final systematic assessment. Extracted data are presented according to the main diagnostic outcome as detailed below. We created a miscellaneous disorders category for studies where multiple diagnoses were reported.

#### Alexithymia in the Treatment of Mood Disorders

Our systematic search identified six studies examining the role of alexithymia in the treatment of mood disorders. Ozsahin et al. (26) conducted the first follow-up study to assess the impact of alexithymia on response to antidepressant treatment and the impact of antidepressant treatment on alexithymia levels in patients affected by major depression (MD). The working hypothesis was to investigate the influence of alexithymia on the short-term outcome of treatment. The study sample included 65 outpatients with MD, 32 with alexithymia, and 33 devoid of alexithymia, who were treated with paroxetine, 20 mg/day for 10 weeks. Over the course of treatment, changes in HAM-D scores were found to correlate significantly with baseline alexithymia levels measured using TAS-20. Conversely, changes in TAS-20 scores failed to correlate with baseline levels of depression. The percentage of responders, defined by a 50% decrease in total HAM-D scores versus baseline, was 21.9% in the alexithymia group and 54.5% in the group without alexithymia. Following treatment, 31.2% of basal alexithymia patients had returned to the category of non-alexithymia patients, obtaining a cut-off score at TAS-20 below the threshold for alexithymia, while alexithymia persisted in 68.8% of cases. Response to antidepressant treatment was observed in 50% of patients who were no longer alexithymic compared to baseline, and in only 9.1% of patients with persistently stable alexithymia. Based on these findings, the authors highlighted the potential of alexithymic features and stability of alexithymia to produce a negative influence on short-term response to antidepressant treatment in patients with MD. Subsequently, Ogrodniczuk et al. (27) conducted a retrospective study to assess the potential role of alexithymia in predicting the persistence of residual symptoms following short-term individual psychotherapy. A sample of 33 outpatients with a diagnosis of unipolar MD was studied. Data relating to all patients selected were extrapolated from a randomized controlled study of two forms of short-term individual psychotherapy (interpretative and supportive); patients all responded positively to psychotherapy, as attested by scores obtained at the Beck Depressive Inventory (BDI) (50% reduction in scores obtained at BDI rating scale and a post-treatment BDI score equal to or lower than 8). Concomitant to psychotherapy, 52% of patients received antidepressant treatment (tricyclics or selective serotonin reuptake inhibitors) for a period of at least 6 weeks. Residual depressive symptoms were observed in 82% of recruited patients. No significant correlations were revealed between baseline levels of depression and levels of alexithymia and individual factors at TAS-20, thus underlining a scarce association between alexithymia and severity of depressive symptoms at baseline. Scores obtained for DIF factor at TAS-20 were predictive of the severity of residual symptoms (measured using BDI) to a considerably higher extent than initial levels of depression and anxiety, type of psychotherapy, and use of antidepressants. The authors concluded that difficulty in identifying feelings may impact on the subject’s ability to effectively benefit from psychotherapy and might consequently be considered a factor underlying the persistence of residual symptoms. The results obtained also support the notion according to which alexithymia and depression are separate constructs, particularly bearing in mind the scarce correlation between baseline levels of depression and scores relating to alexithymia factors. Even when giving due consideration to the effect of initial levels of depression and anxiety, to the form of psychotherapy received and to the use of antidepressants, scores relating to the “Difficulties Identifying Feelings” (DIF) factor of TAS continued to act as a predictor of the severity of residual symptoms. A possible explanation for the association between DIF and residual symptoms might lie in the patient’s inability to identify his or her feelings, thus potentially compromising the ability to effectively share these feelings and emotional problems with the therapist who, faced with a series of vague complaints may not be able to develop an effective treatment plan for the patient. Moreover, the ability of the patient to understand the measures implemented by the therapist relating to his or her feelings may be compromised. Given the importance in psychotherapy of working with feelings and bearing in mind the difficulty of alexithymic patients of sharing their feelings, treatment may not achieve the desired efficacy. Based on the hypothesis of a series of authors who considered alexithymia a stable characteristic capable of hindering a favorable outcome to psychotherapy, Spek et al. (28) conducted a study to assess whether alexithymia was a stable or changeable trait when associated with a change in depressive symptoms, and whether the pre-treatment presence of alexithymia prevented the patient from benefitting from psychotherapeutic treatment. A sample of 129 subjects affected by sub-threshold depression (presence of significant depressive symptoms although not sufficient to meet DSM-IV criteria for a diagnosis of major depression) underwent cognitive behavioral therapy. The type of outcome was defined according to the change in depressive symptoms as measured by BDI, from the pre-treatment to the post-treatment stage and at 1-year follow-up. Changes in depressive symptoms correlated significantly with changes in alexithymia, measured using TAS-20, thus demonstrating how alexithymia is actually a less stable trait than frequently maintained. Baseline alexithymia levels were not indeed correlated with treatment outcome. The authors hypothesized that this finding, at variance with other studies using different forms of psychotherapy, may be related to the possibility that CBT represents a less problematic treatment option in alexithymia patients. Gunther et al. (29) carried out a prospective study to evaluate the association between pre-treatment alexithymia levels (TAS-20) and depressive symptoms at follow-up (7 weeks) following multimodal inpatient treatment. Forty-five patients admitted for acute MD who had taken part in a multimodal treatment program comprising both individual therapy and psychodynamic-interactional oriented group therapy were studied. The majority of patients were also prescribed antidepressant treatment. Of all TAS-20 factors, only scores obtained for the “Externally Oriented Thinking” EOT factor were predictive of the severity of depressive symptoms at follow-up, when measured using both self-administered questionnaires (BDI-II) or clinician-administered scales (HAM-D). A higher baseline score for the EOT factor correlated with more severe depressive symptoms, even following evaluation of potential confounding factors, such as baseline levels of anxiety and depression. Total baseline scores at TAS-20 were predictive for severity of depressive symptoms measured by means of BDI, but not HAM-D. A moderate correlation was moreover revealed between alexithymia (DIF and “difficulties describing feelings” DDF factors) and self-reported depressive symptoms at baseline, in accordance with previous studies reporting a moderate correlation between DIF and DDF, but not EOT, with depression (30). Furthermore, alexithymia was found to be a stable trait, as a significant reduction in scores at BDI-II and HAM-D failed to correspond to a significant change over time in scores obtained at total TAS-20 and factors DIF, DDF, and EOT. The impact of alexithymia in terms of outcome was demonstrated for the EOT factor, but not for DIF and DDF. Several authors maintain that the EOT factor is a fundamental characteristic of alexithymia and emotional ability (31). DIF and DDF factors have been subject to debate due to their sensitivity to a series of biases in assessing the emotional ability of an individual (self-critical response biases, perfectionism, mood-congruent memory biases). The finding of elevated scores for DIF and DDF in depressed patients may indeed correlate not only with a more marked level of alexithymia, but also with a singular predisposition to provide specific answers induced by the state of depression. Conversely, the EOT factor may well be less susceptible to this type of response bias, focusing tendentially on the subject’s preferences and habits, and may result in a more precise indicator of the alexithymic construct in clinical populations. The authors suggest that patients affected by MD with a more externally oriented cognition and lower interest in intrapsychic conflicts may derive less benefit from a multimodal therapeutic approach (including psychodynamic interactional therapy, antidepressant medication, and complementary therapies). Bearing in mind that the program used envisaged a series of therapeutic approaches, it is unclear which aspect of the treatment (group or individual psychotherapy, antidepressant drugs or complementary therapies) may prove less effective in the presence of an externally oriented cognition. In a study conducted by Bressi et al. (32), the authors evaluated the efficacy of Short-Term Psychodynamic Psychotherapy (STPP) using Mentalization-Based Techniques (STMBP) on the clinical outcome of 274 patients affected by MD, focusing on the impact of Reﬂective Functioning (RF) and baseline alexithymia on treatment outcome. The duration of treatment was 40 weeks, and patients were assessed at T0, T1 (after 40 weeks) and T2 (after 1-year follow-up). All patients recruited to the study were receiving antidepressants (SSRIs or SNRIs). Treatment proved effective on both depressive symptoms (HAM-D) and levels of alexithymia. A significant reduction of scores for the EOT factor and, to a lesser extent DIF, was observed between T0 and T1. A further improvement of alexithymia, limited to EOT, was subsequently observed at follow-up. Higher levels of depression at HAM-D and higher levels RF at T0 were predictive of a worse treatment outcome. Alexithymia levels (TAS-20) at T0 displayed no significant impact in terms of outcome. Quilty et al. (33) investigated the role of alexithymia in predicting the therapist- and patient-rated therapeutic alliance and response to psychotherapy (CBT and IPT) in a sample of patients with MD. Seventy-five adults affected by MD were randomized to two treatment arms comprised of weekly individual sessions of CBT or IPT over a period of 16 weeks. Scores obtained at TAS-20 and factor DIF correlated with a negative impact on the patient-rated therapeutic alliance at week 13; at week 13, DIF factor alone displayed a negative impact on therapist-rated therapeutic alliance. Path models supported the hypothesis of a direct negative effect of alexithymia on response to treatment by means of a negative association with therapeutic alliance. More specifically, alexithymia was negatively associated with the alliance which, in turn, was positively associated with the change in depression registered during treatment. Contrary to expectations, scores for the EOT factor negatively correlated with the severity of depression at week 13 (pre-treatment, a rise in EOT corresponded to a decrease in severity of depression) and correlated positively with the change in depression (pre-treatment, a rise in EOT was associated with a more marked change in levels of depression from week 1 to week 13). Data present in literature in line with the hypothesis whereby therapists were negatively influenced by the alexithymic features of their patients were further confirmed by the findings of the present study that highlighted the impact of alexithymia on patient-rated perception of therapeutic alliance, including perception of the understanding and involvement of the therapist. Moreover, the results of the study invite us to reflect on the impacts produced by the different facets of the alexithymic construct in terms of clinical outcome. The study conducted by Quilty et al. (33) revealed a negative prognostic role of alexithymia in general, and of the DIF factor on patient-rated therapeutic alliance, and a positive prognostic role of the EOT factor on depression. The authors underlined the possibility that the DIF factor might impact on fundamental psychotherapeutic activities such as cognitive restructuring, which may be limited to those patients unable to adequately express their feelings. On the other hand, it may be the case that the EOT factor in depressed patients correlates with a decrease in ruminative thinking and, hypothetically, with more adequate adaptive and problem-focused coping strategies. The therapeutic strategies used for the purpose of the study (CBT and IPT), in view of their characteristics as active and objective-oriented treatments, may explain the reduced impact of alexithymia in negatively affecting outcome of the study treatment, at variance with other studies conducted using a different approach (27). All these findings are summarized in **Table 1**.

**Table 1:** Studies investigating the impact of alexithymia on treatment outcome in mood disorders.

Reference	Objectives	Study design	Sample size	Standardized assessment of alexithymia	Treatment	Assessment tools	Diagnosis	Results
Ozsahin et al., (26)	To investigate the influence of alexithymic features on depression treatment outcome, along with the eventual change in alexithymia burden over the course of treatment.	Prospective design with patients evaluated before beginning treatment (T0), and post-treatment (T1) after 10 weeks.	65 (32 alexithymic and 33 non-alexithymic)	TAS-20	Antidepressant (i.e. Paroxetine)	SCID for DSM-IV, HAM-D	MDD	At T1 a positive correlation was described between HAM-D and TAS-20, with a significantly greater HAM-D score reduction among non-alexithymic individuals.
Ogrodniczuk et al. (27)	To investigate the association between alexithymia and residual symptoms among short-term psychotherapy responders in the outpatient setting.	A 20-week RCT including individuals randomly assigned to receive either interpretative or supportive psychotherapy	33	TAS-20 DIF, DDF, EOT	20 weekly sessions of either interpretative or supportive psychotherapy (17/33 concomitantly received antidepressant medication, either a SSRI or a tricyclic; these individuals were equally distributed between the 2 study groups)	Computer assisted SCID I and II for DSM-III R, BID assessed pre- and post-treatment, STAI	MDD	No association was found between baseline alexithymia and baseline depression severity; DIF was significantly associated with residual depressive symptoms.
Spek et al. (28)	To investigate the interplay between alexithymia and CBT outcome at 12 months follow-up	Prospective design with 1-year follow-up	119	TAS-20	CBT psychotherapy	BDI	Subthreshold MDD	Changes in alexithymia were significantly correlated with BDI changes, however no significant association was found between pre-treatment alexithymia and treatment outcome.
Gunther et al. (29)	To study the relationship between alexithymia and symptom severity after a course of	Prospective design with assessments performed after an average of 2 weeks from admission (T0) and at 7 weeks after starting therapy (T1)	45	TAS-20 DIF, DDF, EOT	Psychodynamic interactional psychotherapy (at T1 33/45 individuals were also taking antidepressants)	BDI-II, HAM-D, SCID for DSM-IV, STAI	MDD	Baseline EOT (T0) predicted depressive symptom burden at T1.
Bressi et al. (32)	A 12-month follow-up study exploring the effectiveness of STMBP in MDD (1), the possible correlation among alexithymia and reflective functioning (2), the correlation among clinical variables and their eventual impact on TAS-20 and HAM-D	Prospective design with assessments (GAF, HAM-D, TAS-20) at baseline (T0), after 40 weeks (T1) and at 12 months follow-up (T2)	24	TAS-20	40 weekly session of STMBP; all study participants were taking antidepressant medications (SSRI, SNRI); during the follow-up no medication allowed except for occasional BDZs administration	AAI-RF, GAF, HAM-D	MDD	A reduction in HAM-D and TAS-20 scores was described, along with a negative correlation between RF and TAS-20 score.
Quilty et al. (33)	To test the role of alexithymia in influencing CBT and IPT treatment outcome in MDD affected individuals.	An 16-week RCT with patients randomized either to IPT or CBT (38 to IPT, 37 to CBT)	75	TAS-20 DIF, DDF, EOT at baseline	16 weekly sessions of either IPT or CBT (no antidepressant medication was allowed during the trial)	BDI-II and CALPAS at 3-8 and 13 weeks; SCID for DSM-IV, HAM-D	MDD	A negative correlation was described for EOT and 13-week depression burden; an increased alexithymia level was also associated with lower alliance score

AAI-RF, Adult Attachment Interview-RF; BDZ, benzodiazepine; BID, Beck Depression Inventory; CALPAS, California Psychotherapy Alliance Scale; CBT, cognitive behavioral therapy; DIF, TAS-20 factor 1 Difficulty Describing Feelings; DDF, TAS-20 factor 2 Difficulty Identifying Feelings; DSM-III, Diagnostic and Statistical Manual of Mental Disorders III edition; DSM-IV, Diagnostic and Statistical Manual of Mental Disorders IV-edition; HAM-D, Hamilton Depression Rating Scale; IPT, Interpersonal Therapy; MDD, major depressive disorder; RCT, randomized controlled trial; RF, reflective functioning; SCID, Structured Clinical Interview for DSM-IV; SNRI, serotonin norepinephrine reuptake inhibitor; SSRI, selective serotonin reuptake inhibitor; STAI, Spielberger State Trait Anxiety Inventory; STMBP, Short-Term Psychodynamic Psychotherapy with Mentalization-Based Techniques; TAS-20, Toronto Alexithymia Scale 20 items.

#### Alexithymia in the Treatment of Eating Disorders

Nine studies investigating the role of alexithymia in eating disorders were identified by our systematic search. Schmidt et al. (34) were the first to analyze the impact of alexithymia on response to short-term pharmacological treatment of bulimia nervosa (BN). Forty-one female outpatients with a diagnosis of BN according to DSM-III-R were recruited to a 10-week prospective study of patients receiving fluvoxamine treatment. Alexithymia levels failed to correlate with any of the other variables investigated at either baseline or T1. On completion of treatment, higher TAS scores registered at T1 correlated with a persistently higher number of episodes of binge eating. Overall, post-treatment, no significant change in total TAS scores was registered versus baseline, although the eating pathology displayed a marked improvement. The authors hypothesized that the persistence of eating symptoms and a failure to achieve recovery during treatment might correlate with the presence and persistence of elevated TAS scores. The concomitant absence of a significant change in TAS following treatment, albeit in the presence of an improvement of the eating disorder (ED), appeared to suggest that alexithymia should not be seen as a dimension that correlates exclusively with the ED trend, but rather as an independent feature. Subsequently, de Groot et al. (35) assessed the impact of alexithymia on response to a 9.6-week intensive group psychotherapy program focused on nutrition, body image, management of symptoms, relationships, and family interactions (Toronto Hospital Day Hospital Program for ED). The sample was comprised of 31 women with severe BN according to DSM-III-R, and a control group of 20 healthy subjects (all nurses) was set up. On completion of treatment, patients who were more alexithymic at T1 were also found to be those most affected by both ED symptoms and depression. Although on completion of treatment patients’ mean alexithymia levels remained high - considerably higher than those of healthy controls, overall treatment had produced a positive impact not only on eating symptomatology, but also on alexithymia levels, particularly in a subgroup of patients who had been devoid of binge episodes and vomiting (abstinent) over the 28 days prior to discharge. The authors linked the partial reversibility of alexithymia to a series of factors, including a direct effect of treatment, reduction of associated depressive symptoms, and reduction of eating symptomatology. A cross-sectional study conducted by Beales et al. (36) studied a total of 79 women affected by severe, chronic ED treated with a range of therapeutic options, who were divided into 3 groups — anorexic, bulimic, and recovered — according to EDI-2. Scores obtained at TAS-20 revealed that 65% anorexic, 83% bulimic, and 33% recovered patients were alexithymic, with a significant difference between those presenting with ongoing ED and the group of recovering patients. This led the authors to hypothesize that lower levels of alexithymia may have a potentially relevant impact on the recovery of ED patients. The specific study design prevented assessment of the impact of treatment on alexithymia levels.

Becker-Stoll et al. (37) evaluated 47 female patients with ED diagnosed according to DSM-IV criteria, who attended the Treatment Center for Eating Disorders of the Max Planck Institute of Psychiatry in Munich. A three-phase treatment plan was envisaged: a 4-week outpatient motivation phase, a 4-month day-hospital phase, and a 4-month outpatient follow-up treatment phase. Treatment options included Cognitive Behavioural Therapy (CBT), Interpersonal Therapy (IPT) and Psycho-educational Therapy (PET). During group psychotherapy sessions, art therapy, among others, was used to stimulate the identification and expression of feelings. Post-treatment, both eating symptomatology and alexithymia improved irrespective of diagnosis. The most significant impact produced related to the factor DIF, while EOT was impacted only marginally. Despite evidence of an improvement of alexithymia, patients displayed an ongoing tendency towards alexithymia. No correlation was detected in the study between baseline TAS and outcome variables. Conversely, high TAS scores registered post-treatment, indicating persistent alexithymia, correlated with an increased severity of eating symptoms and a less favorable prognosis. In a subsequent study, Shiina et al. (38) investigated the effectiveness and predictors of drop-out in a treatment program comprised of 10-week outpatient group therapy combining CBT with assertiveness training and self-esteem building therapy (combined group CBT). The final stages of treatment envisaged two role-play sessions during which patients were expected to identify and try to express their feelings. Treatment produced a positive effect on both eating pathology and alexithymia, self-esteem, and social functioning. Baseline alexithymia levels were not predictive of drop-out. In 2007, bearing in mind the limitations of the previous studies, Speranza et al. (39) carried out a long-term naturalistic prospective study with 3-year follow-up to evaluate the impact of alexithymia on the outcome of an outpatient sample of ED patients who underwent a range of therapeutic interventions available in routine clinical practice. The sample was made up of 102 young women affected by severe, chronic ED, 39 of whom with BN and 63 with AN. Exclusion criteria included major depressive episode or ongoing substance or alcohol addiction. Two categories of clinical outcome were envisaged: “favorable outcome” with full remission of eating symptoms at follow-up, and an “intermediate/unfavorable outcome” with persistence of a subsyndromal pattern or full ED diagnosis at follow-up. At 3-year assessment 75% of patients displayed an “intermediate/unfavorable outcome” and 25% “favorable outcome”. The majority of patients had undergone at least one form of therapeutic intervention over the three-year period, mainly psychotherapy (57%) or treatment for depression (40%). DIF factor of TAS-20 was found to be a significant predictor of unfavorable outcome. The predictive power of this factor persisted, although to a lesser extent, in a second predictive model taking into account depressive symptoms, clinical severity of the disorder, and treatment prescribed. Patients experiencing more difficulty in identifying their feelings at baseline were more frequently symptomatic at follow-up, displaying a substantially less favorable clinical course. Tchanturia et al. (40) conducted a cross-sectional study to evaluate a sample of 148 subjects, 105 of whom with either ongoing ED or recovered meeting DSM-IV criteria, in comparison with a group of healthy controls (n = 43) with no family or personal history of mental disorders. Patients with ongoing ED displayed higher levels of alexithymia and social anhedonia versus recovered patients, collocated on an intermediate level, and healthy controls who registered the lowest levels of alexithymia at TAS-20. A significant correlation was revealed between persistence and severity of ED symptoms and higher levels of alexithymia and social anhedonia. This correlation remained even when depression was taken into account in analysis. A study by Balestrieri and colleagues (41) analyzed the factors underlying response to a short-term (10 weeks) psychoeducational outpatient group treatment. The study was conducted on a sample of 98 patients (91% women) with a diagnosis of BED (n=54) and EDNOS (n=44) according to DSM-IV. Each treatment session included nutritional counselling, analysis of thoughts and behaviors correlated with ED, and assertiveness training. On completion of treatment patients displayed an improvement, although of negligible clinical significance, in total TAS scores. Lower levels of alexithymia at baseline were predictive of a higher probability of patients achieving recovery from ED following treatment. Finally, Ohmann et al. (42) studied 29 adolescent girls with AN in a 10-month multimodal group CBT study conducted in an outpatient setting. Treatment provided for nine modules (therapeutic motivation, psychoeducation, individual problem analysis, teaching of problem-solving strategies, soft and communication skills, hedonistic training, elements of awareness, body, and schema psychotherapy) and monthly family sessions. On request, individual CBT sessions were arranged. Three separate patient groups were subsequently obtained on the basis of outcome: good outcome (n=16), bad outcome (n=5), drop-out (n=8). Evaluations made throughout the different treatment stages (3, 6, 9, and 12 months after onset and 1 year after completion of treatment) revealed lower levels of alexithymia in the good outcome group of patients compared to the bad outcome and drop-out groups. Alexithymia was shown to be a factor resistant to change, particularly in patients with a bad outcome, even in the presence of improvement of other clinical variables including BMI, eating habits, mood, social anxiety, personal care, and self-efficacy. These findings are summarized in **Table 2**.

**Table 2:** Studies investigating the impact of alexithymia on treatment outcome in eating disorders.

Reference	Objectives	Study design	Sample size	Standardized assessments of alexithymia	Treatment	Assessment tools	Diagnosis	Results
Schmidt et al. (34)	To investigate alexithymia prevalence among individuals affected by DSM-III defined eating disorders as compared with healthy controls (1), eventual differences in alexithymia prevalence among the included nosological categories (2), alexithymia persistence in these conditions (3), the predicting value of alexithymia for short-term treatment outcome (4)	Combination of cross-sectional study and of a 10-week double blind placebo-controlled trial	(a)173 F cases (93 BN, 55 AN/R, 25 AN/BN); 95 healthy controls (48 F, 47 M). (b)41 F individuals affected by BN	a. TAS-26 b. TAS-26	(b) Fluoxetine vs placebo	a. BITE, BSQ b. BITE, BSQ, HAM-D	AN/RN, AN/BN, BN	(a)Cases had a significantly higher alexithymia prevalence than controls. TAS did not correlate with BMI, BITE, BSQ. (b)TAS at t0 correlated with TAS at t1 but did not correlate to any other variable. TAS at t1 positively correlated with rater-assessed binge.
de Groot et al. (35)	To estimate alexithymia prevalence among women affected by DSM-III defined BN treated in a DH (1), alexithymia relationship with somatic symptoms and depression (2), efficacy of group psychotherapy in reducing alexithymia burden	Prospective study with assessments pre- and post-treatment; case control analysis with a comparison group assessed at 1 point only	31 cases, 20 controls	TAS-26	Psychotherapeutic group focusing on body image, nutrition, family interactions and symptoms management (average duration of treatment 9.6 weeks)	BDI, EDE, EDI	BN	A greater proportion of BN affected individuals presented alexithymia as compared with healthy controls before treatment (t0); post-treatment (t1) there was a significant reduction in alexithymia proportion among BN individuals, but it persisted at a higher level than the comparison group
Beales et al. (36)	To explore the presence of alexithymic features in a selected group of individuals affected by ED and the potential implications of the said features for the primary care setting	Survey	79	TAS-20		EDI-2, 16-PF5,	AN/R, BN and RV	A higher prevalence of alexithymia was found among AN/R and BN groups as compared with the R group; 16PF5 social skills domain negatively correlated with alexithymia
Becker-Stoll et al. (37)	To investigate the potential efficacy of an intensive 4-month intervention program on alexithymia in ED (DSM-IV defined) and the possible alexithymia role in predicting treatment outcome	Prospective design with assessments performed before (t0) and after treatment (t1)	47	TAS-20	A 4-month psychotherapeutic program employing interpersonal, cognitive-behavioral and psychoeducational methods.	EDI	AN, BN, EDNOS	There was a significant reduction in both EDI-2 and TAS-20 (especially DIF) at T1. TAS-20 score at T1 correlated with EDI2 at T1 and with a worse prognostic outlook; there was no significant correlation between TAS at t1 and the recovery state
Shiina et al. (38)	To study the efficacy of a CGCBT for BN affected individuals in the outpatient setting, further exploring the characteristic of individuals failing to respond under such treatment	Prospective design with assessments at the beginning (T0) and at the end of the treatment course (T1)	25	TAS-20	1-h weekly sessions of CGCBT over a 10-week period, including diet psychoeducation, social skill training, self-esteem enhancement, coping training for interpersonal problems	BITE, CGI-C, CGI-S, EDI-2, GAF, HAM-D, RSES	BNP, ANBP, BNNP, EDNOS	Among the 16 individuals that completed the treatment course, at T1 there was a significant reduction in BITE, GAF, EDI-2, RSES scores as compared with T0; mean TAS-20 scores showed a non-significant reduction (p= 0.06). No significant association was found between TAS-20 and treatment outcome.
Speranza et al. (39)	To investigate the influence of alexithymia on treatment outcome in a large sample of ED affected individuals	3-year longitudinal study	102	TAS-20 DIF, DDF, EOT	Due to the naturalistic study design no treatment was specifically recommended; of the total sample 57% was undergoing psychotherapy, 40% was on antidepressants	MINI, BDI-13, CGI-S, PSRS	ED	At the 3-year follow-up assessment 76 patients were judged to have an unfavourable prognosis with DIF being a significant predictor of a negative outcome
Tchanturia et al. (40)	To explore the complex interplay between ED, social anhedonia and alexithymia	Observational study	148	TAS-20 DIF, DDF, EOT	Due to the observational nature of the study, no specific treatment was recommended	SCID for DSM-IV, DASS, EDE-Q, RSAS	AN, BN	A positive correlation was described between social anhedonia and alexithymia
Balestrieri et al. (41)	To explore the efficacy of a 10-week psychoeducational group program among BED and EDNOS affected individuals, and the persistence of its eventual benefits	A 1-year follow-up study with assessments before treatment (T0), after treatment (t1) and at 1-year follow up (T2)	98	TAS-20	10-week psychoeducational group including nutritional interventions and thoughts related to eating disorder along with assertiveness training. After the first 10-weeks, those individuals still satisfying ED criteria were involved in a further extension protocol comprising 8 additional monthly sessions	EDI-2, EDI-SC, HADS	BED, EDNOS	A lower or absent alexithymia level was associated with a higher likelihood of responding to treatment
Ohmann et al. (42)	To explore emotional problems of young individuals affected by AN and undergoing GCBT	A 12-month follow-up study with assessments before treatment (T0), during treatment (at 3 and 6 months, T1 and T2, respectively), and post-treatment (T3) after 12 months	29	TAS-26	GCBT focusing on psychoeducation, schema psychotherapy, communication skill training, problem analysis, therapeutic motivation, hedonistic training, problem solving (5 individuals were concomitantly treated with antidepressants)	ASW, BDI, JTCI, MDI, YSR	AN	Only two patients described themselves as not alexithymic. Alexithymia presented a non-significant trend toward improvement in responding individuals.

16PF5, Sixteen Personality Factor Questionnaire 5th Edition; AN/RN, Anorexia Nervosa/Restrictive subtype; AN/BN, Anorexia Nervosa/Bulimic subtype; NBP, Anorexia Nervosa Binge-eating/Purging type; ASW, Assessment of Self-Efficacy; BDI-13, Beck Depression Inventory 13 items version; BITE, Bulimic Investigatory Test; BMI, Body Mass Index; BNNP, Bulimia Nervosa Non-Purging type; BNP, Bulimia Nervosa Purging type; BSQ, Body Shape Questionnaire; CGCBT, Combined Group Cognitive Behavioral Therapy; CGI-C, Clinical Global Impression Change; CGI-S, Clinical Global Impression Severity; DIF, TAS-20 factor 1 Difficulty Describing Feelings; DDF, TAS-20 factor 2 Difficulty Identifying Feelings; DSM-III, Diagnostic and Statistical Manual of Mental Disorders III-edition; DSM-IV, Diagnostic and Statistical Manual of Mental Disorders IV-edition; ED, eating disorders; EDE-Q, Eating Disorder Examination Questionnaire; EDI-2, Eating Disorder Inventory-2; EDI-SC, Eating Disorder Inventory-Symptom Checklist; EDNOS, eating disorder not otherwise specified; EOT, TAS-20 factor 3 Externally Oriented Thinking; GCBT, Group Cognitive Behavioral Therapy; HADS, Hospital Anxiety and Depression Scale; HAM-D, Hamilton Depression Rating Scale; HC, healthy control; JTCI, Junior Temperament and Character Inventory; MDI, Marburg Diagnostic Inventory; MINI, Mini International Neuropsychiatric Interview; PSRS, Psychiatric Status Rating Scale; RAN, recovered anorexia nervosa; RSAS, Revised Social Anhedonia Scale; RSES, Rosenberg Self-Esteem Scale; RV, recovered; SPS, Social Phobia Scale; SIAS, Social Interaction Anxiety Scale; TAS-20, Toronto Alexithymia Scale-20; YSR, Youth Self Report.

#### Alexithymia in the Treatment of Anxiety Disorders

A study carried out by Rufer et al. (43) is the sole study to have specifically investigated the role of alexithymia as a predictor of outcome in the treatment of anxiety disorders. In the context of a naturalistic study, the authors assessed the impact of alexithymia on the outcome of a short-term cognitive-behavioral group therapy (CBGT) lasting 5 weeks. Fifty-five consecutive outpatients with panic disorder (PD), 58% of whom without agoraphobia and 42% with agoraphobia, were enrolled. Forty percent of patients presented with comorbidity of one or two Axis I disorders, mainly major depression. Patients underwent CBGT and were assessed at baseline, post-treatment, and at follow-up 6 months after completion of treatment. Thirty-five percent of patients were on concomitant antidepressant treatment. Baseline alexithymia levels were not predictive of the outcome of CBGT either post-treatment or at 6-month follow-up. Conversely, the presence of comorbidity with Axis I disorders was predictive of outcome post-treatment but not at follow-up. A comparison between treatment completers and non-completers and follow-up completers and non-completers failed to reveal any significant differences in alexithymia levels. Over the course of treatment, a reduction in scores obtained for total TAS-20 and factors DIF and DDF of TAS was observed, but not for factor EOT, which remained substantially stable. A tendency towards improvement of alexithymia during treatment persisted even after verifying the potential influence of depression which strongly correlated with alexithymia pre-treatment. The authors suggest that failure to detect a negative impact of alexithymia on outcome of CBGT treatment may be attributable to the efficacy of this psychotherapeutic approach on the type of patient studied, not focused specifically on the degree of insight nor on verbal interventions, but rather on behavioral “experiments” such as exposure (43). The opportunity for alexithymic patients with PD to experience new emotional events during exposure sessions might motivate patients to modify a series of dysfunctional beliefs and behaviors, in turn capable of impacting positively on their anxiety symptoms. These studies are listed in **Table 3**.

**Table 3:** Studies investigating the impact of alexithymia on treatment outcome in diverse psychiatric disorders.

Reference	Objectives	Study design	Sample size	Standardized assessments of alexithymia	Treatment	Assessment tools	Diagnosis	Results
Kosten et al. (44)	To explore the complex interplay between alexithymia and treatment outcome in PTSD	An 8-week double blind RCT with randomization to either imipramine, phenelzine or placebo	57	APRQ	8-week course of either imipramine, phenelzine or placebo	IES	PTSD	Alexithymia level was significantly associated with a worse treatment outcome
Bach and Bach (45)	To evaluate pre-treatment alexithymia as a potential outcome predicting factor among individuals affected by SD, along with the assessment of alexithymia level heterogeneity among different diagnostic categories	Prospective design with assessments performed at baseline and at 2-years follow-up	30	TAS-26	Integrated behavioral therapy over a minimum of 8 weeks including exposure, group cognitive therapy, muscle relaxation and assertiveness training	WI, SCID, SCL-90R	SD, PD, HY, USD	A non-significant correlation between higher pre-treatment TAS-26 score and USD persistence at follow-up was described
McCallum et al. (46)	To explore the predicting value of alexithymia and PM	Reanalysis of two previously published clinical trials	251	TAS-20 DIF, DDF, EOT	Either 12 weeks of weekly STGT or 20 weeks of STIT	PMAP	CG, MDD, AVO, DEP, BPD, DST, OCD, PAR	A modest portion of improvement variance was linked to alexithymia and PM in both treatment group.
Rufer et al. (47)	To test the predicting value of alexithymia among OCD patients undergoing CBT	Prospective design with assessment before and after treatment	39	TAS-20 DIF, DDF, EOT	Multimodal CBT (25 individuals received concomitant antidepressant)	Y-BOCS, HAM-D	OCD	Alexithymia level did not predict treatment outcome
Rufer et al. (48)	To investigate alexithymia outcome predicting value for OCD in the long term	A 6-year prospective design with assessments before, after treatment and at 6 years follow-up	34	TAS-20 DIF, DDF, EOT	Multimodal CBT (25 individuals received concomitant antidepressant)	Y-BOCS, HAM-D	OCD	Alexithymia level did not predict treatment outcome at follow-up
Grabe et al. (49)	To explore alexithymia persistence in the inpatient setting and its influence on the outcome	Prospective analysis with assessments at T0 at baseline, T1 at 4 weeks and at discharge T2	297	TAS-20	Treatment duration varied from 8 up to 12 weeks administered in the inpatient setting and included: 3 weekly sessions of psychodynamic STGT and 1 weekly session of individual psychotherapy; daily art, sport, movement and relaxation therapy (medications were administered as needed)	SCL-90R, GSI	AUD, MDD, ADD, SFD, DIS, ED, PED	Higher levels of psychological stress were described among alexithymic individuals as compared with non-alexithymic individuals; alexithymia was not associated with a higher likelihood of early withdrawal from therapy, nor with a higher degree of treatment resistance. Nonetheless, a higher post-treatment GSI was described among alexithymic
Leweke et al. (50)	To investigate baseline alexithymia influence on treatment outcome in an inpatient setting	Prospective design with a 4 or an 8-week treatment course depending on the underlying condition	480	TAS-26 DIF, DDF, EOT, RD	Multimodal treatment including psychodynamic oriented individual psychotherapy, associated with art, group body and music therapy; pharmacotherapy was offered as needed	SCL-90R, GSI	DD, ADS, ASD, PTSD, ADJ, SFD, ED	Alexithymia was associated with a small risk for worse outcome as compared with non-alexithymic.
Löf et al., (51)	To investigate the complex interplay between alexithymia, self-image and treatment outcome among BPD undergoing MBT.	Prospective design with a 12-month treatment course; assessments were performed at baseline, at 6, 12, and 18 months.	75	TAS-20 DIF, DDF, EOT, RD	Multimodal treatment comprising individual and group MBT; pharmacotherapy was administered as needed.	DSHI-9, KABOSS-S, MINI, RQ, SASB, SCID-II, SCL-90-R, ZAN-BPD	BPD	No correlation was described between treatment outcome and alexithymia.
Rufer et al. (43)	To test alexithymia predictive value on treatment outcome among PD individuals receiving a course of CBT (1), and the eventual change of alexithymia over time (2).	Prospective	55	TAS-20 total score, DIF, DDF, EOT	5 sessions of GCBT (19 patients received concomitant pharmacotherapy)	MINI, BDI, PAS-20	PD with and without agoraphobia	Alexithymia level decreased over time, but it did not predict GCBT outcome. The EOT factor remained more stable over time.
Ogrodniczuk et al. (45)	To test the potential efficacy of a group therapy among outpatient psychiatric users, and the impact eventual alexithymia changes in interpersonal functioning	Prospective 2-year observational study with assessments at baseline, post-therapy and at 3 months follow-up	68	TAS-20 DIF, DDF, EOT	5 weekly sessions of group therapy for 3 months	BDI, IIP-28	AD, DD, PED	Alexithymia level was associated with greater interpersonal difficulties at follow-up, with higher alexithymia changes corresponding to greater improvement in interpersonal functioning
McMain et al. (52)	To test the relationship between treatment outcome and specific changes in emotion processes and problem-solving	A subset analysis of an RCT	80	TAS-20 DIF, DDF, EOT	Either multiple weekly sessions of DBT (individual and group therapy) or GPM (combined psychodynamic and pharmacotherapy)	DABS, SCID-I, SCL-90-R, IIP-64, LIWC	BPD	No significant correlation was described between alexithymia level and treatment outcome; changes on the DDF significantly predicted IIP improvements
Terock et al. (53)	To study the relationship between alexithymia, SD and their eventual influence on the outcome	Prospective analysis with assessments at admission and discharge	716	TAS-20 DIF, DDF, EOT	6-8 weeks of psychodynamic oriented therapy with cognitive behavioral elements (pharmacotherapy was offered as needed)	SCL-90R, GSI, TCI	AD, AUD, ED, PED, SFD	The DIF was the only factor in the TAS-20 predicting treatment outcome.
Probst et al. (54)	To explore the complex interplay between alexithymia, therapeutic alliance and treatment outcome in MSD	Reanalysis of a 12-week RCT	83	TAS-20 DIF, DDF, EOT	12 sessions of weekly PIT	SCID for DSM-IV, HAQ, PHQ-9, PCS	MSD	No significant relationship was described between alexithymia, therapist alliance and treatment outcome when controlling for depression burden
McGillivray (55)	To study the potential influence of alexithymia on treatment outcome	Prospective study with assessments performed at the beginning and at the end of the treatment course	61	TAS-20 DIF, DDF, EOT	Integrated group therapy CBT-based	DASS-42	AD, MD, SFD, PED	No significant correlation was described between alexithymia and treatment outcome either at baseline or after treatment
Zorzella et al. (56)	To test the influence of alexithymia on treatment outcome among women with a history of sexual abuse	Prospective study with assessments were performed at baseline (T0), post BRG (T1), post WRAP (T2)	51	TAS-20 DIF, DDF, EOT	4-6 weekly sessions of a group therapy (BRG) followed by a further 8-week course of multimodal trauma therapy WRAP (e.g. CBT, Psychoeducation, IPT)	CTQSF, PTSDC, DES, IIP, MMPI, PSI, WAI-S	PTSD	A significant correlation was described between alexithymia improvements at T1 and T2 and changes in dissociation, PTSD and IP at the same timepoints

AD, anxiety disorders; ADD, adjustment disorder; ADJ, adjustment disorder; APRQ, Alexithymia Provoked Response Questionnaire; ASD, acute stress disorders; AUD, alcohol use disorder; BDI, Beck Depression Inventory; BPD, borderline personality disorder; BRG, Building Resources Group; CBT, cognitive behavioral therapy; CG, complicated grief; CTSFQ, Childhood Trauma Questionnaire Short Form; DABS, Derogatis Affects Balance Scale; DASS, Depression Anxiety Stress Scale; DD, depressive disorders; DIF, TAS-20 factor 1 Difficulty Identifying Feelings; DDF, TAS-20 factor 2 Difficulty Describing Feelings; DIS, dissociative disorders; DSHI, Deliberate Self-Harm Inventory-9; DSM-III, Diagnostic and Statistical Manual of Mental Disorders III; DST, dysthymia; ED, eating disorders; EOT, TAS-20 factor 3 Externally Oriented Thinking; GSI, Global Severity Index; GPM, general psychiatric management; HAM-A, Hamilton Anxiety Rating Scale; HAM-D, Hamilton Depression Rating Scale; HAQ, Helping Alliance Questionnaire; HC, healthy control; HY, hypochondria; IES, Impact of Events Scale; IIP-64, Inventory of Interpersonal Problems-64 items; IIP-28, Inventory of Interpersonal Problems-28; IPT, Interpersonal Therapy; KABOSS-S, Karolinska Borderline and Symptoms Scales; LIWC, Linguistic Inquiry and Word Count; MBT, Mentalisation-based Therapy; MD, mood disorders; MDD, major depressive disorder; MINI, Mini-International Neuropsychiatric Interview for DSM-IV; MMPI, Minnesota Multiphasic Personality Inventory; MSD, Multisomatoform Disorder; PCS, Physical Component Summary from the SF-36; PD, panic disorder; PED, personality disorders; PHQ-9, Patient Health Questionnaire; PIT, Psychodynamic-Interpersonal Psychotherapy; PM, psychological mindedness; PMAP, Psychological Mindedness Assessment Procedure; PSI, Problem-Solving Inventory; PTSD, posttraumatic stress disorders; PTSDC, Post-Traumatic Stress Disorder Checklist; RD, reduced daydream; RQ, Relationship Questionnaire; SASB, Structural Analysis of Social Behavior; SCID-II, Structured Clinical Interview for DSM-IV Axis-II disorders; SCL-90, Symptoms Checklist 90R; SD, somatization disorder; SED, self-directedness; SFD, somatoform disorder; STGT, Short-Term Group Therapy; STIT, Short-Term Individual Therapy; STP, short-term psychotherapy; TAS-20, Toronto Alexithymia Scale 20 items; TCI, Temperament and Character Inventory; USD, Undifferentiated Somatoform Disorder; WAI-S, Working Alliance Inventory, short form; WI, Whiteley Index; WRAP, Women Recovering From Abuse Program; Y-BOCS, Yale-Brown Obsessive-Compulsive Scale; ZAN-BPD, Zanarini Rating Scale for Borderline Personality Disorder.

#### Alexithymia in the Treatment of Post-Traumatic Stress Disorders

Two studies on Post-Traumatic Stress Disorder (PTSD) were identified in our systematic review. The first study to investigate the role of alexithymia as predictor of treatment response in PTSD dates back to 1992 (44). The authors analyzed a sample of 57 Vietnam veterans affected by PTSD according to DSM-III. The impact of alexithymia on response to treatment was evaluated in the context of an 8-week randomized controlled double-blind trial with two treatment arms (imipramine and phenelzine) and 1 placebo arm. All participants underwent concomitant individual psychotherapy based on a short-term psychodynamic approach. Alexithymia levels were assessed using the APRQ. Lower levels of alexithymia were predictive of a better outcome for avoidance items (emotional numbing, distance from others, and efforts to avoid thinking about the traumatic event) on the Impact of Events Scale (IES), irrespective of the severity of trauma. This finding was only relevant for placebo-treated subjects receiving psychotherapy. No impact was observed for on intrusion items of the IES or in pharmacologically-subjects receiving psychotherapy. As all three treatment groups received psychotherapy in addition to the pharmacological agent, the results of the study suggest that psychotherapy alone may not be effective in reducing avoidance symptoms in alexithymic patients with PTSD. More recently, Zorzella et al. (56) examined the role of alexithymia in terms of improvement of trauma-specific difficulties experienced before and after trauma therapy in women with a history of childhood abuse. Date were collected from 167 women enrolled in the Women Recovering from Abuse Program (WRAP), an 8-week multimodal treatment program comprising a series of therapeutic options, both group and individual, specifically devised for women with a history of severe childhood trauma. Contrary to expectations, baseline alexithymia levels were not predictive of the magnitude of change registered post-treatment in PTSD symptoms, dissociation, and interpersonal issues. Higher baseline levels of alexithymia (TAS-20) prior to any form of treatment were found to be predictive of increased severity of PTSD, higher degree of dissociation, and additional interpersonal difficulties. At the same time, improvement in alexithymia levels over the course of treatment correlated significantly with better treatment response in terms of symptoms of PTSD, levels of dissociation, and interpersonal difficulties. The methodology applied in the study does not enable a causal relationship to be established between these variables. These studies are summarized in **Table 3**.

#### Alexithymia in the Treatment of Personality Disorders

Two studies analyzed the impact of alexithymia on the treatment of personality disorders. The first study to analyze the implications of alexithymia on treatment outcome in personality disorders (PD) was carried out by McMain et al. (51). The authors conducted an exploratory study aimed at investigating the relationship between specific emotional processes and cognitive problem-solving processes and treatment outcome in subjects with borderline personality disorder (BPD). Patients were recruited from an extended sample of subjects enrolled in a randomized controlled study to compare the clinical effectiveness and cost-effectiveness of a yearly program of Dialectical Behavior Therapy (DBT) or General Psychiatric Management (GPM), a multimodal outpatient treatment comprised of individual psychodynamic therapy, case management, and symptom-targeted medication management. The sample selected for the study was made up of 80 subjects, mainly women, all of whom treatment completers, in line with the specific aims of the study. The majority of participants presented with one or more comorbidities with other Axis I and/or Axis II mental disorders (major depressive disorder, anxiety disorders, and substance use disorders). Assessments were carried out at baseline (pre-treatment) and then every 4 months during the course of active treatment. An analysis was conducted to ascertain whether changes observed during treatment (identification of emotions, ability to describe emotions, externalizing, verbalization of positive emotions, affect balance, perceived cognitive problem solving) impacted on treatment outcome. Assessments were made bearing in mind the working alliance, a consolidated outcome predictor. Improvements observed over the course of treatment relating to the ability to identify, describe, and fully experience emotions were associated with a more favorable treatment outcome (symptom distress and interpersonal functioning). Löf et al. (51) conducted a longitudinal naturalistic study aimed at assessing the effectiveness of Mentalization-based treatment (MBT) and psychiatric and psychological moderators of outcome in a sample of 75 patients with BPD. Evaluations were made at baseline and 6, 12, and 18 months after start of treatment. Treatment proved effective on borderline symptoms and on general psychiatric symptoms, suicidality, self-harm, alexithymia, particularly difficult in identifying feelings, and self-image. Alexithymia, similarly to severity of BPD and, in general, to psychological moderators, revealed no predictive effect in terms of outcome. These studies are summarized in **Table 3**.

#### Alexithymia in the Treatment of Obsessive-Compulsive Disorders

Our systematic review identified two studies assessing the role of alexithymia in obsessive compulsive disorder (OCD) treatment. Rufer et al. (47) evaluated absolute and relative stability of alexithymia in response to treatment and the predictive power of alexithymia on treatment outcome in patients with OCD. Forty-two patients affected by OCD were recruited to the study, hospitalized, and subjected to intensive multimodal CBT for a mean period of 70 days. Treatment provided for both individual and groups sessions, as well as pharmacological treatment with antidepressants, largely fluvoxamine, for some of the patients. Although treatment proved effective on obsessive compulsive symptoms and associated depressive symptoms, no significant changes were observed from pre- to post-treatment for total TAS-20 or factors DIF and EOT. A decrease in levels of factor DDF alone was registered during treatment, and a relative stability of alexithymia emerged as a personality trait rather than a state-dependent phenomenon in obsessive-compulsive patients. Neither alexithymia pre-treatment nor other variables examined (depression and pharmacological treatment) appeared to significantly predict response to multimodal CBT in OCD. Bearing in mind the limitations of the previous study which, being devoid of a follow-up period, had not enabled the long-term course of alexithymia or its predictive power on the long-term outcome of treatment to be established, Rufer et al. (48), followed on from this work to conduct a long-term study with a 6-year follow-up period. Thirty-four of the 42 patients enrolled in the previous study (47) were recruited and, thus, reassessed 6 years after their hospitalization. In addition to confirming the relative long-term stability of alexithymia, the study also confirmed the absence of a predictive power of alexithymia levels, both pre- and post-treatment, on the long-term outcome of OCD. On the other hand, the decrease in alexithymia levels observed at follow-up (total TAS-20, DIF, and DDF) may have been implicated in protecting some patients against a worsening of obsessive-compulsive symptoms during the follow-up period. These findings are illustrated in **Table 3**.

#### Alexithymia in the Treatment of Somatoform Disorders

The only study to date to have specifically investigated the role of alexithymia as predictor of treatment outcome in somatoform disorders (SD) was carried out by Probst et al. (54). In reanalyzing data from a multicenter randomized, controlled trial on brief psychodynamic-interpersonal therapy (PIT) for patients with multisomatoform disorders, the authors addressed the issue of whether alexithymia moderated the association between therapeutic alliance and outcome of PIT and the implications of the depression variable on these potential effects. 107 patients affected by multisomatoform disorder with pain as predominant symptom were randomized to PIT (duration 12 weeks) and 104 patients to the control condition (enhanced medical care, EMC). Only the subsample randomized to PIT was considered for the purpose of analysis, based on the singular relevance of the therapeutic alliance in PIT compared to the control condition. All patients met the criteria for a somatic disorder according to DSM-5. The outcome was based on self-reported physical quality of life 9 months after completion of treatment. Pre-treatment alexithymia and therapeutic alliance post-treatment and at 9-month follow-up were rated by both patients and therapists. Neither alexithymia nor therapeutic alliance correlated with PIT outcome. On considering patient-rated therapeutic alliance, alexithymia was not found to moderate the associations between alliance and outcome. Conversely, when considering therapist-rated therapeutic alliance, alexithymia was found to significantly moderate the relationship between alliance and treatment outcome. A stronger alliance in the therapists’ perspective was beneficial for the outcome only in patients with very high scores at TAS-20, and therefore with very high levels of alexithymia. The importance of the therapist’s perception of a good alliance with the patient is underlined, also in view of an improved outcome when treating patients with alexithymia. As demonstration of the complex interaction between alexithymia and depression, the significance of alexithymia as a moderator of the alliance-outcome link was lost when pre-treatment levels of depression as covariate to the moderation model. These results are presented in **Table 3**.

#### Alexithymia and Its Impact on Treatment in Samples With Diverse Psychiatric Disorders

A series of studies assessed the impact of alexithymia on treatment response analysis samples with diverse diagnoses. Bach & Bach (57) investigated the potential role of alexithymia in predicting long-term treatment outcome in 30 patients admitted to hospital for somatoform (36.7%) and panic disorders (63.3%). Thirteen of the 19 patients affected by panic disorder presented with an additional diagnosis of somatoform disorder. In all subjects, the presence of functional somatic symptoms, mainly cardiorespiratory, gastrointestinal, and neurological symptoms constituted the main reason underlying request for treatment. All patients underwent integrative behavior therapy with both group and individual sessions over a period of hospitalization of no less than 8 weeks. The study envisaged diagnostic assessment prior to hospitalization and 2 years after discharge. Assessment at 2-year follow-up revealed how patients who met the criteria for undifferentiated somatoform disorder had presented with higher baseline levels of alexithymia (TAS-26) versus patients who had gone into remission from the somatoform disorder and those who had never met the criteria for somatoform disorder. No other baseline or follow-up diagnosis was found to correlate significantly with baseline levels of alexithymia. Higher levels of alexithymia at baseline were therefore predictive of a persistent somatization, irrespective of other variables relating to psychopathology, socio-demographics or severity of the disorder, thus demonstrating the potential role of alexithymia in predicting relapse in the long-term and a less favorable response to treatment in somatizing patients. The presence of alexithymic characteristics, and therefore, a difficulty for patients to identify and share their feelings, might make these subjects more susceptible to communicating through use of bodily sensations, an aspect that could complicate the process of recovery from their illness. Several years later, McCallum et al. (46) explored the impact of alexithymia and psychological mindedness (PM) as predictors of outcome in 4 forms of short-term psychotherapy. Data were extrapolated from two comparative trials of interpretive therapy versus supportive therapy. The first trial provided for a once-weekly session of short-term group therapy over a period of 12 weeks in 107 outpatients with complicated grief. The second trial involved 144 psychiatric outpatients with a mixed diagnosis, who underwent once-weekly short-term individual therapy for a total of 20 weeks. Seventy-one percent of patients met the criteria for an Axis I diagnosis, with a higher prevalence of major depressive disorder; fifty-five percent of patients were affected by an Axis II disorder; 38% of patients received a diagnosis of both Axis I and Axis II disorders. In the two trials, both alexithymia (TAS-20) and PM were found to be predictive of outcome. Higher levels of PM and lower levels of alexithymia were associated with a more favorable response to the four forms of therapy, with the additive effect of the two variables on outcome. In supportive individual therapy, the DDF factor of TAS-20 was inversely associated with an improvement on General Symptoms. In the short-term individual therapy trial, the DIF factor was found to be inversely associated with improvement on General Symptoms and Social-Sexual Maladjustment; the EOT factor was inversely associated with improvement on Social-Sexual Maladjustment. One clinical implication of these findings may lie in the hypothesis whereby, generally speaking, patients with lower levels of alexithymia and a higher PM may prove better suited to psychotherapy, both of an interpretative and supportive nature. Likewise, patients displaying high levels of alexithymia and low levels of PM pre-treatment may experience an improvement of these characteristics following psychotherapy, and thus benefit from treatment in the same way as subjects who approach therapy with low levels of alexithymia and high levels of PM. With regard to the impact of alexithymia of the different forms of interpretative and supportive therapy, non-alexithymic patients were generally found to benefit from both supportive and interpretative treatment. Contrary to expectations, and bearing in mind the specific deficits of subjects with alexithymia, although alexithymia was not found to interfere with the ability of patients to gain benefit from individual interpretative therapy, it was however seen to interfere with their ability to benefit from individual supportive treatment, at least in terms of improvement of symptoms. The authors suggested that this finding might be explained by the attitude of the therapist during supportive therapy; indeed, by avoiding the exploration and interpretation of an association between symptoms and underlying feelings, the therapist could further exacerbate the initial difficulty of the alexithymic subject to identify and share his or her feelings. Although the findings of the study underline the predictive value of alexithymia on treatment outcome, they however fail to provide sufficient elements to allow this variable to be considered of use in opting for one specific therapeutic approach over another.

Grabe et al. (49) assessed a total of 414 consecutive psychiatric patients admitted for hospital treatment, 297 of whom had been followed up at 4 weeks (T1) and 8-12 weeks (T2) after discharge. Patients underwent psychodynamic group therapy in a naturalistic setting over a period of 8-12 weeks. Pharmacological treatment was offered when clinically indicated. Art therapy, sport therapy, relaxation therapy, body, and movement therapy were available on a daily basis. Patients admitted to psychotherapy were affected by depressive disorders, anxiety and adjustment disorders, somatoform disorders, eating disorders, and comorbid alcohol-related disorders and personality disorders. Subjects with alexithymia at T0 displayed a higher Global Severity Index at Symptom Checklist-90, and consequently increased psychopathological distress at T0, T1, and T2. Over the course of treatment, the group with alexithymia displayed a significant improvement over time of alexithymia levels and at individual factors at TAS-20, at variance with the very slight reduction in TAS scores manifested by the patients without alexithymia. Levels of alexithymia in patients who had suspended treatment within the first 4 weeks were no higher than those of patients who had continued treatment, thus highlighting a lack of interference of the alexithymia variable on treatment compliance. Alexithymic features were therefore found predictive of a worse long-term outcome in this study. In the context of an extended study conducted on 480 psychiatric patients from a series of diagnostic categories (eating disorder, depressive disorder, anxiety disorder, acute or post-traumatic stress disorder, somatoform disorder), Leweke et al. (50) assessed alexithymia as outcome predictor of psychodynamic-oriented multimodal inpatient therapy. The treatment program included both individual and group psychodynamic-oriented therapy, art and music therapy, and pharmacological treatment as indicated. Both short-term and long-term treatments were envisaged, with duration ranging from 4 to 8-12 weeks. A high baseline level of alexithymia (TAS-26) was found to be a significant predictor of treatment outcome only in patients with somatoform disorder, with a marked association with the DDF factor, whereby subjects registering higher scores at factor DDF displayed a less favorable evolution of symptoms.

Ogrodniczuk et al. (45) evaluated 68 psychiatric outpatients with heterogeneous psychopathological issues who were enrolled in a comprehensive group therapy program organized in group sessions 5 days a week for a total of 12 weeks. Patients were assessed at baseline, post-therapy, and at 3-month follow-up. Alexithymia levels, in particular factor DIF at TAS-20, were found to improve significantly during treatment. A reduction in alexithymia levels during treatment and throughout the follow-up period were significantly associated with an improvement in interpersonal issues both during treatment and the follow-up period, i.e. in the long-term. The findings of the study confirmed the hypothesis according to which a positive change in alexithymia levels may contribute towards an improved treatment outcome, particularly with regard to interpersonal functioning. In the light of this evidence, the authors highlighted the feasibility of considering alexithymic traits as changeable features, thus focusing treatment come on the optimal management of the same with the aim of producing a positive impact on treatment outcome. In 2015, Terock et al. (53) investigated the impact of alexithymia and self-directedness (SD) on general psychopathology and on treatment outcome in a sample of 716 consecutively admitted day-clinic outpatients with alcohol/drug dependence and abuse (8.7%), depressive disorders (84.4%), anxiety and somatoform disorders (18.3%), eating disorders (0.6%), and personality disorders (20,5%). Routine treatment provided for psychodynamic-oriented psychotherapy with the inclusion of elements of CBT. Duration of the treatment program ranged from 6 to 8 weeks according to patients’ individual needs. The program included sessions of art therapy, group therapies, and individual psychotherapy, in addition to pharmacological treatment when clinically indicated. Both baseline alexithymia levels (TAS-20) and SD levels were found to be significant predictors of psychopathological stress at baseline at the Global Severity Index of SCL-90 (GSIT0). SD, but not alexithymia levels, proved to be a significant predictor of treatment outcome as measured by GSI at T1. The DIF factor of TAS was the only strong predictor of GSI both at T0 and T1, thus constituting the sole alexithymic factor capable of predicting treatment outcome. Changes in levels at TAS-20, together with changes in SD were predictive of GSI at T1. The results obtained highlight the higher impact of the SD variable on treatment outcome compared to alexithymia. More recently, McGillivray et al. (55) evaluated the role of alexithymia on treatment outcome in a naturalistic group therapy setting. Sixty-one psychiatric outpatients affected by mood disorders (54.1%), neurotic, stress-related, and somatoform disorders (19.7%), disorders of adult personality and behavior (14.8%), schizophrenia, schizotypal, and delusional disorders (9.8%) and behavioral syndromes associated with physiological disturbances and physiological factors (1.6%) were enrolled in the study and assessed both pre- and post-treatment. Treatment lasted for approximately 8 weeks and was based on a CBT approach. Baseline alexithymia levels, in relation to both total scores obtained at TAS-20 and to individual factors, was not found to be a significant predictor of treatment outcome (change in psychological distress) once baseline psychological distress was controlled for. In the course of treatment, a small, albeit significant, reduction in mean alexithymia scores at TAS-20, and reduction in alexithymia levels during treatment, proved to be a significant predictor of a decrease in psychological distress during treatment. The authors hypothesized that absence of a correlation between baseline alexithymia levels and treatment outcome might be associated with the efficacy of the implemented group cognitive behavioral therapy on the alexithymia dimension, as demonstrated by the findings of the study. All these findings are summarized in **Table 3**.

## Discussion

Over the years increasing debate has been focused on the issue of whether, and if so to what extent, alexithymia should be considered a stable personality trait or a phenomenon linked to psychopathological status, and whether it is capable of predicting treatment outcome. However, in spite of the increasingly frequent findings confirming the clinical significance of alexithymia and its correlation with a wide range of physical complaints and mental disorders, studies conducted to investigate, in the context of psychiatric disorders, the implications of alexithymia as a predictor of treatment outcome are still numerically scarce and methodologically limited. More and more frequently in the field of psychiatry, the finding of sub-optimal responses to treatment requires an increasingly personalized treatment plan focused on the individual and his or her characteristics, problems, and specific needs, rather than merely on his or her illness. The need to improve treatment outcome in the case of mental disorders implies a growing urgency to identify outcome predictors, both generally and in specific categories of patients and for specific therapeutic approaches. The reasons underlying the hypothesis whereby alexithymia is purported to be significantly implicated in terms of impact on the outcome of mental disorders are numerous and include a negative influence of alexithymia on clinical expression of the disorder and on response to therapeutic intervention, its correlation with other disorders and pathological behaviors, in addition to treatment choices made by the clinician and the clinical setting. Of note, a recent study showed that distinct clinical and sociodemographic characteristics, such as lower educational level, high rates of psychiatric comorbidity as well as of cardiological comorbid disorder, were associated with alexithymia in mood disorder patients (16). These findings point to alexithymia as a relevant determinant of adverse outcomes in psychiatric disorders, underlying its potential in the implementation of personalized care. For the purpose of the present review, a total of 30 studies were selected and analyzed. Eating disorders represented the mental disorders in which alexithymia fostered increasing interest as a predictor of outcome. These were followed by mood disorders (depressive disorders), personality disorders (borderline personality disorders), post-traumatic and obsessive-compulsive disorders, and lastly, somatoform (multisomatoform disorders) and anxiety disorders (panic disorder). Overall, seven studies focused on the analysis of mixed patient samples with a series of psychiatric diagnoses. Almost all studies selected featured a longitudinal prospective design, 1 study had a retrospective design (27), and 2 studies a cross-sectional design (36, 40). The studies invariably focused on evaluation of the predictive impact of alexithymia on the outcome of different forms of group or individual psychotherapy (cognitive behavioral therapy, psychodynamic therapy, interpersonal therapy, psychodynamic-interpersonal therapy, psychoeducational therapy, mentalization-based treatment, rhythmic movement therapy, dialectical behavior therapy) conducted in a series of treatment settings (doctor’s surgery, day-hospital, hospital). Three studies alone assessed the impact of alexithymia primarily on the outcome of pharmacological treatment (imipramine and phenelzine versus placebo; fluvoxamine; paroxetine) (26, 34, 44). It should however be highlighted that even when the primary treatment was psychotherapy, a percentage of patients also received concomitant pharmacological treatment. In many cases the treatment program provided for an integrated approach comprising both individual and group psychotherapeutic and pharmacological measures, in addition to the use of a series of psychotherapeutic techniques. This prevented the identification of the degree of impact of specific approaches or measures on alexithymia levels and insight into the type of interventions impacted to a greater extent by alexithymia, particularly in terms of outcome. In the majority of cases, samples recruited were relatively small, scarcely representative, and featured a marked heterogeneity with regard to patient characteristics (outpatients, inpatients, acute or chronic, with varying degrees of severity and alexithymia levels), diagnostic groups considered, and psychiatric comorbidities. Moreover, in view of the scarce number of patients assessed in the majority of studies selected, the additional impact of missing data both post-treatment and during the follow-up period, if envisaged, should be taken into account. A short longitudinal observation period was implemented in numerous studies, limiting assessment of alexithymia levels to pre- and post-treatment. Only a few studies implemented a medium to long-term follow-up (28, 32, 37, 39, 42, 43, 45, 48, 49, 51, 54, 57), the presence of a control group of healthy individuals (34, 35, 40) or comparison between the different therapeutic approaches used (27, 33, 44, 46, 52, 54). Moreover, in the studies selected, the alexithymia dimension was detected solely by means of self-reported assessments which, in view of the issues addressed and the high risk of confounding by other pathological variables, may not be sufficiently reliable in measuring the true entity of alexithymic involvement. Finally, certain studies Overall, although the results obtained should be read and interpreted bearing in mind the numerous limitations of studies available to date, the data provided largely correlate lower baseline and/or post-treatment levels of alexithymia and/or improvement in alexithymia levels over the course of treatment, with a less favorable treatment outcome on the mental disorders considered.

A smaller number of studies, although substantial (8 out of 30 studies), failed to acknowledge a predictive role for alexithymia in terms of treatment outcome (28, 32, 38, 43, 47, 48, 51, 54). In the study by Kosten et al. (44), a double-blind, randomized controlled trial of patients with PTSD, higher levels of alexithymia were found to be predictive of a worse outcome with regard to avoidance symptoms, although only in subjects treated with placebo and psychotherapy. This finding led the authors to hypothesize that when given alone, psychotherapy may not be effective in reducing avoidance symptoms in PTSD patients with alexithymia. In the study conducted by Leweke et al. (50) higher baseline levels of alexithymia were a significant predictor of treatment outcome only in individuals affected by somatoform disorder, showing a particularly strong correlation with the DDF factor, with higher scores indicating a less favorable evolution of symptoms in these subjects.

When the studies included did not report correlations between levels of alexithymia and treatment outcome, to justify this lack of confirmation the authors mentioned: efficacy of treatment on alexithymia, and therefore improvement of alexithymia in the course of treatment; increased feasibility and adaptability of a series of therapeutic approaches in patients with alexithymia; a different impact of alexithymia in relation to the diagnostic group considered. In the context of the naturalistic study conducted by Rufer et al. (43) the authors attributed failure to detect a negative impact of alexithymia on treatment outcome to the type of psychotherapy approach based on behavioral “experiments” used in the study, maintaining that this approach may have elicited a change in alexithymic features, thus impacting positively on symptoms of anxiety.

Throughout the majority of studies analyzed, the impact of alexithymia on treatment outcome was assessed on the basis of the association between baseline alexithymia levels and outcome variables post-treatment and, where relevant, at follow up. In other cases, the correlation between improvement in alexithymia levels during treatment and outcome variables was analyzed (45). In yet others, both aspects were taken into account, at times yielding discordant findings (55, 56). In the study carried out by Zorzella et al. (56) in a sample of patients with PTSD, an improvement in alexithymia levels over the course of treatment was found to correlate significantly with a better response to treatment, in relation to both PTSD symptoms and levels of dissociation and interpersonal difficulties. This was in line with the findings of McGillivray et al. (55) who investigated a sample of psychiatric outpatients with different diagnoses, all of whom treated with CBT. In some studies, the evaluation was limited to alexithymia levels post-treatment and outcome variables (36, 40). Despite a failure to detect a correlation between correlation between baseline alexithymia levels and treatment outcome, other studies highlighted how higher levels of alexithymia post-treatment, indicating persistent alexithymia, correlated with an increased severity of the disorder post-treatment, and accordingly, with a lower response to treatment (34, 37).

Most of the studies examined confined their evaluation to the global alexithymia construct, implying that total baseline or post-treatment TAS scores or improvements in total TAS over the course of treatment correlated with outcome variables. A smaller number of studies investigated the different facets of alexithymia and assessed their impact as predictor of outcome. The study conducted by Speranza et al. (39) found the DIF factor to be the best predictor of an unfavorable outcome, being negatively correlated in particular with clinical improvement of patients with severe or chronic eating disorders. This was in line with the findings of Ogrodniczuk et al. (27) and Terock et al. (53). Accordingly, a difficulty in identifying feelings may, hypothetically, impinge on the ability of the individual to gain effective benefit from psychotherapy. Conversely, in a study in hospital inpatients affected by major depression, Gunther et al. (29) detected a predictive role for the severity of depressive symptoms at follow-up solely for the EOT factor. Indeed, higher EOT scores at baseline correlated with more severe depressive symptoms, even once the potential influence of confounding factors, such as baseline levels of anxiety and depression had been accounted for. The authors suggested that such a multimodal therapeutic approach might not be effective in depressed patients with a more marked externally oriented cognition and decreased interest in intrapsychic issues.

At variance with the observations made by these authors, Quilty et al. (33) reported a positive prognostic role for the EOT factor. The authors underlined the possibility that in depressed patients the EOT factor might be associated with a decrease in ruminative thinking, and therefore, with a more adaptive and problem-focused coping strategy. In addition, McCallum et al. (46) revealed how higher levels of psychological mindedness and lower levels of alexithymia at DIF and EOT factors were associated with a more generally positive response to treatment, with an additive effect of the two variables on outcome. Based on the above findings, and despite the current paucity of available data, the observation of a varying predictive impact across the different dimensions of alexithymia, in addition to the diverse impacts of treatment on the various factors, suggests that in future studies it may prove more informative and useful to implement a multidimensional approach to alexithymia. A further fundamental aspect, in the light of which the results obtained in the studies examined should be construed and interpreted, relates to the role of confounding factors. Only a few of the studies examined took into consideration the impact of variables, such as depression, clinical severity of the disorder, psychiatric comorbidities, and treatments received. In some cases, significance of the predictive power of TAS and related factors persisted, although to a lesser extent, even when the impact of depression, clinical severity of the disorder, and treatments received had been taken into account (39). In yet others, the correlation of results obtained for the confounding variables resulted in a loss of significance for the association alexithymia and treatment outcome (54). In the study by Probst et al. (54), the sole study to have specifically assessed the role of alexithymia as outcome predictor in the treatment of somatoform disorders, alexithymia was initially found to significantly moderate the correlation between therapist-rated therapeutic alliance and treatment outcome, although this significance was subsequently lost on accounting for the levels of depression present pre-treatment. With regard to the effect of specific treatments on alexithymia levels, although this aspect was not included among the specific aims of this review, it may be helpful to briefly refer to these. Indeed, several authors assessed the impact of an improvement of alexithymia in the course of treatment on the outcome of the same treatment. Moreover, in some cases failure to detect a predictive power of alexithymia on treatment outcome was attributed by the authors to the particular efficacy of the treatment options adopted on alexithymia. It should however be highlighted that even when a decrease in alexithymia levels was observed post-treatment, the finding that levels frequently continued to be elevated, invites us to reflect on the actual clinical effectiveness of these changes, underlining the need to identify types of treatment capable of producing a significant impact on the alexithymia dimension (35, 37). Furthermore, although the finding that an improvement in alexithymia levels over the course of treatment was largely correlated with a better treatment outcome is certainly of interest, it should be viewed as a preliminary finding which, particularly in view of the methodological limitations of the studies, does not allow any conclusions to be drawn as to possible causality. An additional limitation concern the use, in some studies, of the older 26-item version of the TAS, which might have impacted the reliability in the assessment of alexithymia. Undoubtedly, the increasingly frequent findings reporting alexithymia as a trait which is, at least in part, modifiable, appear encouraging, and should motivate us to undertake more extensive and methodologically valid studies aimed at identifying the forms of treatment capable of impinging more effectively on the alexithymia dimension, thus resulting in an increased probability of impacting on treatment outcome. A considerable number of studies examined observed over the course of treatment an improvement of varying significance at TAS, with a tendentially greater efficacy of treatments providing for specific intervention on feelings (37, 38). At the same time, the marked heterogeneity of treatment strategies adopted and the methodological limitations of the studies are such as to prevent the generalization and comparison of available data.

Crucially, therefore, particular focus should be placed on identifying which types of treatment and elements involved may prove of greater efficacy in patients with alexithymia. Treatment options encouraging the identification, sharing, and understanding of feelings would appear to be more effective in reducing alexithymia (58). Additionally, these patients appear to prefer, and obtain greater benefit from, a group therapeutic setting (58). A group context provides these patients with an opportunity to observe and copy other members of the group who are able to express their feelings more effectively, to gather feedback on their method of communication, to be encouraged to share their feelings with the others in the group, to witness the watering down of their “emotional arousal” with a lower risk of somatic expression, or to be, as needed, mere observers (58). Several authors have highlighted how patients with alexithymia may be poorer responders to classic medical treatments and to psychotherapy, particularly psychodynamic psychotherapy (27). Indeed, in patients with high levels of alexithymia their emotional experiences may prevent the formulation of a fully aware symbolic and verbal elaboration in the course of treatment. This aspect, together with a scarce interest for introspection, might prevent patients with alexithymia from benefitting from psychotherapeutic interventions based on the above activities (48). In a study by McCallum et al. (46), contrary to expectations, while alexithymia was not found to interfere with patients’ ability to benefit from individual interpretative therapy, it did however interfere with their ability to benefit from individual supportive therapy, at least in terms of improvement of symptoms. The authors suggest that this finding could be explained by the attitude of the therapist during supportive therapy who, by avoiding the exploration and interpretation of an association between symptoms and the underlying feelings, might contribute toward maintaining the initial deficit of the alexithymic subject in identifying and describing his or her feelings to the therapist. It would not therefore seem to be appropriate to maintain that patients with alexithymia are necessarily reluctant to undergo psychotherapeutic treatment (59). Likewise, the opinion according to which patients with alexithymia are poorly suited to psychodynamic approaches has to date found little support, although it is assumed that individuals with high levels of alexithymia may gain greater benefit from psychodynamic approaches involving a more active and empathetic role of the therapist, elements of supportive therapy, and a good therapeutic alliance (58). In the study conducted by Speranza et al. (60) patients were found to have been treated differently by their therapists, both on a quantitative and qualitative levels, based on their alexithymic profile. Accordingly, the authors hypothesize that the clinician might incorrectly view this type of subject as being less suited to psychotherapy (60). It has moreover been demonstrated how high levels of alexithymia may elicit negative reactions in the therapist, as well as negative interpersonal processes with controtransferal feelings of frustration or boredom, which might hinder the achievement of a good therapeutic alliance, and thus contribute to a less favorable outcome of treatment (59).

## Conclusions

To conclude, although the results obtained should be scrutinized and interpreted bearing in mind, the marked limitations of the studies published to date, the available data tend largely to correlate low baseline, and/or post-treatment levels of alexithymia and/or an improvement in levels of alexithymia over the course of treatment, with a more favorable outcome of the treatment of the mental disorders considered. However, the presence of numerous discordant findings prevents us from drawing any firm conclusions as to the actual impact produced by alexithymia on the treatment of psychiatric disorders both in general and in specific diagnostic groups. Likewise, at the current state of the art, we are still far from being able to draw conclusions in favor of specific treatment protocols demonstrating a greater efficacy in this type of patients. It is conceivable, however, that the analysis of large datasets containing longitudinally collected information on measures of alexithymia, with innovative analytical methods, including machine learning, could lead to more accurate estimates of the predictive power of this construct in terms of response to treatments. Further studies should be undertaken on more extensive and homogeneous patient populations, including controlled studies and a comparison between different forms of treatment. Moreover, in analyzing the observations made, due consideration should be given to potential confounding factors, and to a multidimensional analysis of alexithymia and an objective assessment of the alexithymic construct performed.

## Author Contributions

FP drafted the first version of the manuscript, performed the assessment of the included manuscripts, and oversaw the systematic search. MM contributed to the writing of the first draft, performed the systematic search, and designed the study. PP performed the systematic search and contributed to the writing. BC critically revised the paper, designed, and oversaw the study. All authors have seen and approved the submitted version of this paper.

## Conflict of Interest

The authors declare that the research was conducted in the absence of any commercial or financial relationships that could be construed as a potential conflict of interest.

The reviewer SC declared a past co-authorship with several of the authors, MM and BC, to the handling editor.
