# Supplementary material for: The Impact of Alexithymia on Treatment Response in Psychiatric Disorders: A Systematic Review
Source: Front Psychiatry. 2020 Apr 16;11:311. doi: 10.3389/fpsyt.2020.00311 (PMC7177022; doi:10.3389/fpsyt.2020.00311)
Supplement: Supplementary file 1 [file Table_1.docx]

**Supplementary Table.** Quality assessment of the included studies with Newcastle – Ottawa scale: cohort and case control studies

Cohort studies

| Reference | Representativeness of the exposed cohort | Selection of the non-exposed cohort | Ascertainment of exposure | Demonstration that outcome of interest was not present at start of study | Comparability of cohorts on the basis of the design or analysis | Assessment of outcome | Was follow-up long enough for outcomes to occur | Adequacy of follow up of cohorts | Total score (/9) |
| --- | --- | --- | --- | --- | --- | --- | --- | --- | --- |
| Kosten et al., 1992 | Yes | Yes | No | NA | Yes | Yes | No | Yes | 5 |
| Bach et al., 1995 | No | NA | Yes | NA | NA | Yes | Yes | No | 3 |
| Degroot et al., 1995 | Yes | No | Yes | NA | No | No | No | Yes | 3 |
| Beales et al., 2000 | No | NA | No | NA | NA | No | No | No | 0 |
| McCallum et al., 2003 | Yes | NA | Yes | NA | NA | Yes | No | Yes | 4 |
| Ozsahin et al., 2003 | Yes | NA | Yes | Yes | NA | Yes | No | Yes | 5 |
| Becker-Stoll et al., 2004 | Yes | NA | No | NA | NA | Yes | No | Yes | 3 |
| Ogrodniczuk et al., 2004 | Yes | NA | Yes | NA | NA | Yes | No | Yes | 4 |
| Rufer et al., 2004 | Yes | NA | Yes | NA | NA | Yes | No | Yes | 4 |
| Shiina et al., 2005 | Yes | NA | No | NA | NA | Yes | No | Yes | 3 |
| Rufer et al., 2006 | Yes | NA | Yes | NA | NA | Yes | Yes | No | 4 |
| Speranza et al., 2007 | Yes | NA | Yes | NA | NA | Yes | Yes | No | 4 |
| Grabe et al., 2008 | Yes | NA | Yes | NA | NA | Yes | No | Yes | 4 |
| Spek et al., 2008 | No | NA | Yes | NA | NA | No | Yes | No | 2 |
| Leweke et al., 2009 | Yes | NA | Yes | NA | NA | Yes | No | No | 3 |
| Löf et al., 2010 | Yes | NA | Yes | NA | NA | Yes | No | Yes | 4 |
| Rufer et al., 2010 | Yes | NA | Yes | NA | NA | Yes | Yes | Yes | 5 |
| Ogrodniczul et al., 2012 | Yes | NA | No | NA | Yes | Yes | No | Yes | 4 |
| Tchanturia et al., 2012 | Yes | Yes | Yes | NA | Yes | Yes | NA | NA | 5 |
| Balestrieri et al., 2013 | No | NA | Yes | NA | NA | Yes | Yes | Yes | 4 |
| McMain et al., 2013 | No | NA | Yes | NA | Yes | Yes | Yes | Yes | 6 |
| Ohmann et al., 2013 | Yes | NA | No | NA | NA | Yes | Yes | No | 3 |
| Terock et al., 2015 | Yes | NA | Yes | NA | NA | Yes | No | Yes | 4 |
| Gunther et al., 2016 | Yes | NA | Yes | NA | Yes | Yes | No | Yes | 5 |
| Bressi et al., 2017 | No | NA | Yes | NA | NA | Yes | Yes | Yes | 4 |
| Probst et al., 2017 | Yes | NA | Yes | NA | NA | Yes | Yes | Yes | 5 |
| Quintly et al., 2017 | No | NA | Yes | No | Yes | Yes | No | Yes | 4 |
| McGillivray et al., 2018 | No | NA | No | NA | NA | Yes | No | No | 1 |
| Zorzella et al., 2019 | Yes | NA | No | NA | NA | Yes | No | No | 2 |

Case control studies

| Reference | Case definition | Representativeness of the cases | Selection of controls | Definition of controls | Comparability of cases and controls | Ascertainment of exposure | Same method of ascertainment for cases and controls | Non-Response rate | Total score (/9) |
| --- | --- | --- | --- | --- | --- | --- | --- | --- | --- |
| Schmidt et al., 1993 | Yes | Yes | No | Yes | No | No | No | No | 3 |
